# Multilokuläre mukokutane Leishmaniose mit Perforation des anterioren Nasenseptums: eine seltene Differenzialdiagnose

**DOI:** 10.1007/s00106-026-01730-8

**Published:** 2026-01-30

**Authors:** Valentin Burkhardt, Daniel Hornuss, Siegbert Rieg, Christoph Becker, Andreas Knopf, Manuel Christoph Ketterer

**Affiliations:** 1 Klinik für Hals- Nasen- Ohrenheilkunde, Universitätsklinikum Freiburg, Medizinische Fakultät, Albert-Ludwigs-Universität Freiburg, Killianstraße 5, 79106 Freiburg, Deutschland; 2 Klinik für Innere Medizin II, Abteilung Infektiologie, Universitätsklinikum Freiburg, Hugstetter Str. 55, 79106 Freiburg, Deutschland

**Keywords:** Leishmaniose, Reisekrankheit, Nasenseptumperforation, Tropenmedizin, Infektionskrankheiten, Leishmaniasis, Traveler’s diseases, Nasal septum perforation, Tropical medicine, Infectious diseases

## Abstract

Die Leishmaniose umfasst kutane, mukokutane und viszerale Formen, verursacht durch Protozoen der Gattung Leishmania. Endemisch ist sie vor allem in Süd- und Mittelamerika sowie im Mittelmeerraum, während sie in Europa sehr selten auftritt. Die mukokutane Leishmaniose kann Nasen- und Mundschleimhäute betreffen. Nachfolgend dargestellter Fall verdeutlicht die Bedeutung interdisziplinärer Zusammenarbeit: Es zeigten sich kutane und mukokutane Herde mit Nasenseptumperforation. Eine enge Kooperation zwischen HNO-Heilkunde, Infektiologie und Tropenmedizin bei seltenen Erkrankungen im Fachgebiet ist entscheidend, um eine zeitnahe und wirkungsvolle Therapie zu ermöglichen.

## Abstract (en)

Leishmaniasis, caused by protozoa of the genus *Leishmania*, comprises visceral, cutaneous, and mucocutaneous forms. It is mainly endemic in South and Central America as well as the Mediterranean region, while being very rare in Europe. Mucocutaneous leishmaniasis may affect the nasal and oral mucosa, and the current case highlights the importance of interdisciplinary collaboration: both cutaneous and mucocutaneous lesions with nasal septum perforation were present. Close cooperation between the departments of otorhinolaryngology, infectious diseases, and tropical medicine are key to ensuring rapid and sufficient treatment.

## Anamnese

Eine 59-jährige Frau aus Deutschland stellte sich in der infektiologischen Ambulanz aufgrund multipler unregelmäßig konturierter Hautulzera mit leicht erhabenem, hyperkeratotischem Randsaum vor. Die Läsionen waren in den letzten Wochen an verschiedenen Stellen aufgetreten, darunter an Wange und Kieferwinkel rechts, Unterarm, Knie, Gesäß und Zeigefinger jeweils links. Außerdem klagte sie über Schmerzen und eine seit einigen Wochen behinderte Nasenatmung. Kopfschmerzen und nasale Schmerzen bestanden seit vier Monaten. Das Auftreten von Epistaxis wurde verneint. Zusätzlich berichtete die Patientin über eine Epiphora seit einigen Wochen.

Nebendiagnostisch berichtete die Patientin von einer ausgeheilten Hepatitis-C-Infektion vor einigen Jahren sowie von einer endoprothetischen Hüftoperation im Jahr 2021. Weitere Nebendiagnosen und die Einnahme von Dauermedikation wurden verneint. Die Patientin berichtete, im Jahr 2021 ein Haus in Costa Rica gekauft und in den letzten drei bis vier Jahren große Teile Süd- und Mittelamerikas bereist zu haben, darunter Kolumbien, Bolivien und Peru.

## Befund

**Abb. 1:**
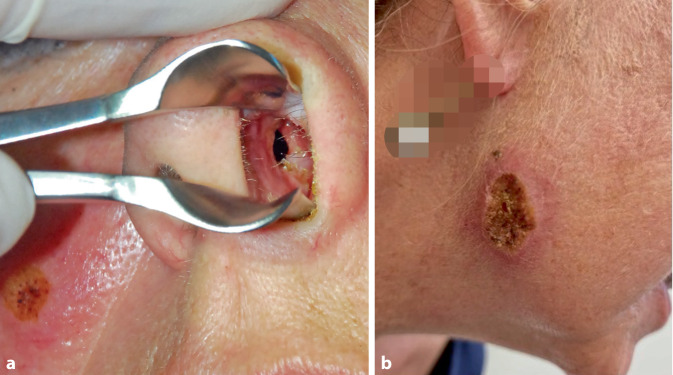
**a **Perforation des Nasenseptums und **b **Darstellung des fazialen Ulkus am Kieferwinkel rechts.

In der klinischen Untersuchung zeigten sich rundliche erythematöse, teils verkrustete Hautulzera an Wange und Kieferwinkel rechts und im Bereich der zuvor beschriebenen Körperstellen (Abb. 1). Die HNO-Untersuchung ergab ein perforiertes Nasenseptum sowie borkig belegte Nasenschleimhaut der linken Seite, einschließlich der unteren Nasenmuschel (Abb. 1, 2 und 3). Die Schleimhäute der Mundhöhle, Pharynx und Larynx zeigten keine Auffälligkeiten.

**Abb. 2:**
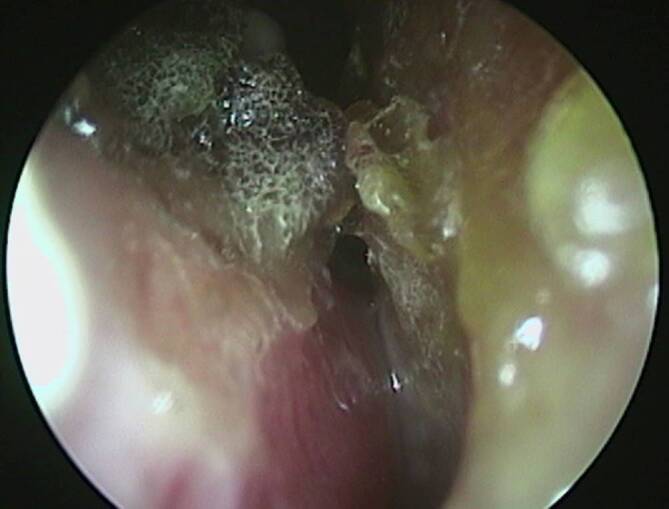
Endoskopische Einsicht in die Nasenhaupthöhle links. Sichtbar sind flächig ausgeprägte Krustenbildungen, welche das Septum und die untere Nasenmuschel bedecken.

**Abb. 3:**
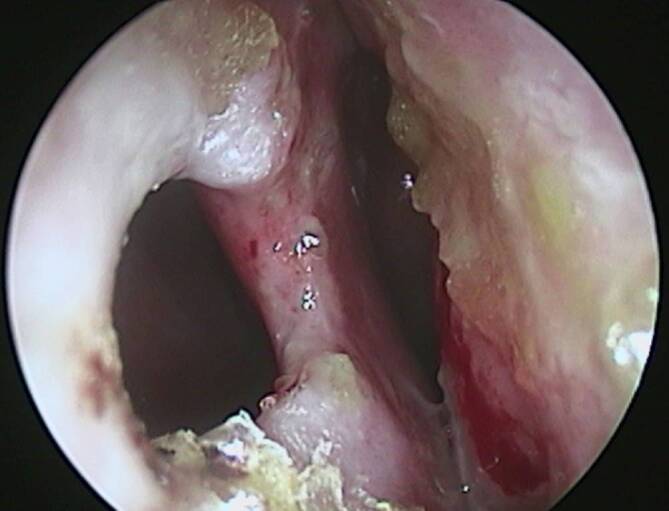
Nach Entfernung der Krusten wird eine Perforation des anterioren Nasenseptums sichtbar.

## Diagnostik und Diagnosesicherung

**Abb. 4:**
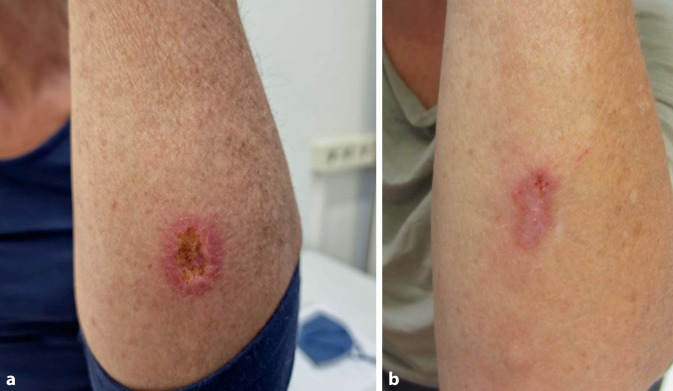
Kutane Läsion des linken Unterarms (**a **prätherapeutisch, **b **posttherapeutisch).

Im Anschluss an die klinischen Untersuchungen erfolgte zur weiteren Abklärung die Entnahme von Blutproben sowie bakteriellen und mykologischen Abstrichen. Ein Serologietest auf humanes Immundefizienzvirus war negativ. Die Routine-Laboranalyse zeigte eine leichtgradige Thrombozytose (394 Tsd./μl), jedoch keine weiteren pathologischen Befunde. Leukozyten und CRP waren ebenfalls normwertig. Die histopathologische Aufarbeitung einer Hautbiopsie der linken Unterarmhaut nahe des Olekranons (Abb. 4) ergab Amastigoten sowie molekulargenetisch den Nachweis von Leishmania panamensis, womit die Diagnose einer mukokutanen Leishmaniose gesichert war.

## Therapie und Verlauf

Die Patientin wurde stationär auf der Bettenstation der Infektiologie aufgenommen und erhielt eine intravenöse Therapie mit liposomalem Amphotericin B (3–4 mg/kgKG) über sieben Tage, was zu einer schnellen Ausheilung der mukokutanen Läsionen führte. Sie konnte zehn Tage nach stationärer Aufnahme wieder entlassen werden. Bei einer klinischen Nachuntersuchung vier Wochen nach der Therapie waren die Hautläsionen gut abgeheilt, jedoch bestand weiterhin eine Nasenseptumperforation sowie eine laborchemische Eosinophilie (0,46 Tsd./μl). Eine zweite Nachuntersuchung vier Monate nach Therapieende zeigte ebenfalls reizlose Narben im Bereich der zuvor bestehenden Hautläsionen bei einer persistierenden Nasenseptumperforation.

## Diskussion

Die Leishmaniose ist eine vektorübertragene Erkrankung, die in drei klinische Manifestationen eingeteilt wird: kutane Leishmaniose (CL), viszerale Leishmaniose (VL) und mukokutane Leishmaniose (MCL). Erreger sind Protozoen der Gattung Leishmania spp., übertragen durch Sandmücken (Phlebotominae) [1].

VL tritt weltweit auf, vor allem durch Leishmania donovani, seltener durch Leishmania infantum. Bei kutanen und mukokutanen Formen wird zwischen der „Alten Welt“ (Europa, Afrika, Asien) und der „Neuen Welt“ (Mittel- und Südamerika) unterschieden. Die klassische CL ist typisch für die „Alte Welt“, während in der „Neuen Welt“ sowohl CL als auch MCL auftreten. Immunsupprimierte Patienten können disseminierte Formen mit Schleimhautbefall entwickeln. Laut WHO war die Leishmaniose 2018 in 92 Ländern endemisch, mit jährlich rund 1 Mio. neuen CL- und 0,2–0,4 Mio. VL-Fällen [2, 3]. Die meisten Fälle treten in Südamerika auf, während in Europa nur wenige pro Jahr gemeldet werden. Zwischen 2005 und 2020 wurden insgesamt 8367 Fälle humaner Leishmaniose an die WHO gemeldet, welche in 69 % einer VL und in 31 % einer CL entsprachen [4]. Aktuelle Daten für Deutschland gibt es aufgrund der fehlenden Meldepflicht an das Robert Koch-Institut nicht.

Eine rechtzeitige eingeleitete Diagnostik und Diagnosestellung der mukokutanen Leishmaniose ist entscheidend, um schwerwiegende Krankheitsverläufe mit persistierenden Funktionseinschränkungen zu verhindern. Unbehandelt kann die Leishmaniose durch Destruktionen des Gewebes, Infektionen und Kachexie auch letal verlaufen. Trotz ihrer Seltenheit in nichtendemischen Ländern kann sie durch zunehmende Reisetätigkeit auch hier auftreten und wird in frühen Stadien oft nicht erkannt.

MCL verursacht destruktive Läsionen der Schleimhäute von Mund, Pharynx und Larynx, in bis zu zwei Dritteln der Fälle auch der Nasenschleimhaut [5]. Die Ausprägung reicht von kleinen Ulzerationen bis zu Septumperforationen und Destruktion der Nasenarchitektur. Die MCL kann infolge direkter, hämatogener oder lymphogener Ausbreitung aus der CL hervorgehen [6].

Der Krankheitsverlauf hängt stark von der Immunantwort des Wirts ab. Viele Infektionen bleiben asymptomatisch, die Parasiten können jedoch persistieren und zu einem späteren Zeitpunkt reaktiviert werden [7]. Die Diagnostik und Diagnosestellung kann anspruchsvoll sein, da CL in Ausprägung, Größe und Dauer der Läsionen variiert und VL andere Erkrankungen wie Tuberkulose, Malaria oder Lymphome imitieren kann [3]. Andere Differenzialdiagnosen einer Nasenseptumperforation, z. B. granulomatöse Erkrankungen wie die Granulomatose mit Polyangiitis oder ein Substanzmissbrauch von Kokain, sollten ebenfalls bedacht werden [7]. Die PCR-DNA-Amplifikation gilt als Goldstandard zur Speziesdifferenzierung, während histologische Giemsa-Färbungen weniger sensitiv sind [8]. Leitlinien empfehlen eine Probenentnahme durch HNO-Ärzte in befallenen Regionen bei Verdacht auf MCL. Serologische Tests sind für CL und MCL nicht geeignet und sollten nur bei VL erwogen werden, wenn Biopsien negativ oder nicht möglich sind [9, 10].

Bei unklaren Hautläsionen sollten CL und MCL daher differenzialdiagnostisch berücksichtigt werden. Die Erhebung einer gezielten Reiseanamnese bei unklaren Hautläsionen, wie im vorliegenden Patientenfall, ist entscheidend für die Diagnose. Disseminierte Verläufe von CL, MCL und VL treten vor allem bei Patienten mit komorbider HIV-Infektion oder Immunsuppression auf [10]. Obwohl bis 2000 in Deutschland kaum Fälle dokumentiert waren [11], können Faktoren wie Reisen, Migration, Haustiere (Hunde können als Reservoir und Überträger der Leishmaniose dienen) und Immunsuppression auch hier das Risiko erhöhen.

Die Leitlinie zur Diagnose und Therapie der Leishmaniose der IDSA/ASTMH (Infectious Diseases Society of America/American Society of Tropical Medicine and Hygiene) empfiehlt bei immunkompetenten Patienten ohne Nachweis einer Hochrisiko-Leishmania-Spezies für ML häufig lediglich eine lokale Kontrolle ohne Therapie [10]. Bei Infektionen mit Spezies aus Hochrisikogebieten sollte jedoch eine systemische Therapie erfolgen, um schwerwiegende Komplikationen wie beispielsweise eine Destruktion der nasalen Knorpelarchitektur zu vermeiden. Standardtherapeutika sind pentavalente Antimonverbindungen, Amphotericin B und Pentamidin [5]. Bei Antimonresistenz, die sehr selten vorkommt, zeigt eine Kombinationstherapie mit Pentoxifyllin vielversprechende Ergebnisse. Auch Miltefosin ist als orales Therapeutikum eine Alternative zur Behandlung der MCL [12]. Amphotericin B führte im vorliegenden Fall zu schneller Heilung. Nach abgeschlossener systemischer Therapie können rekonstruktive Eingriffe zum Verschluss des Nasenseptums mit mukosalen Verschiebelappen oder mit Ohr- oder Rippenknorpel erforderlich werden.

Im deutschsprachigen Raum sind keine weiteren Fälle mit Nasenseptumperforationen bei MCL beschrieben. Andere Kasuistiken beschreiben Schwellungen und Ulzerationen der Lippen und Schleimhäute des Hartgaumens sowie der Nasenschleimhäute mit oberflächlichen Ulzera [13–15], jedoch keine derart ausgeprägten kartilaginären Destruktionen des Nasengerüsts.

## Fazit für die Praxis

- Eine frühzeitige Diagnostik durch zielgerichtete interdisziplinäre Zusammenarbeit zwischen HNO-Ärzten, Infektiologen und Tropenmedizinern ist entscheidend, um eine rechtzeitige Diagnosestellung der Leishmaniose zu gewährleisten.
- Hierfür sind die Kenntnis relevanter Risikofaktoren (bspw. Reiseanamnese und Immunsuppression) und das Wissen um die Leishmaniose entscheidend.
- Zudem ist die interdisziplinäre Zusammenarbeit ein wesentlicher Bestandteil einer optimalen Therapie und kann dazu beitragen, schwerwiegende Verläufe inklusive gravierender Komplikationen wie beispielsweise der Destruktion der nasalen Knorpelarchitektur zu verhindern.
